# Toward ultrafast soft x-ray spectroscopy of organic photovoltaic devices

**DOI:** 10.1063/4.0000214

**Published:** 2024-01-19

**Authors:** Douglas Garratt, Mary Matthews, Jon Marangos

**Affiliations:** 1 Stanford PULSE Institute, SLAC National Accelerator Laboratory, Menlo Park, California 94025, USA; 2 Physics Department, Imperial College London, UK

## Abstract

Novel ultrafast x-ray sources based on high harmonic generation and at x-ray free electron lasers are opening up new opportunities to resolve complex ultrafast processes in condensed phase systems with exceptional temporal resolution and atomic site specificity. In this perspective, we present techniques for resolving charge localization, transfer, and separation processes in organic semiconductors and organic photovoltaic devices with time-resolved soft x-ray spectroscopy. We review recent results in ultrafast soft x-ray spectroscopy of these systems and discuss routes to overcome the technical challenges in performing time-resolved x-ray experiments on photosensitive materials with poor thermal conductivity and low pump intensity thresholds for nonlinear effects.

## I. INTRODUCTION

Development of new materials and devices for the conversion of solar energy into electricity and fuels is crucial for reducing our reliance on fossil fuels. Organic photovoltaics (OPVs) offer an alternative to silicon-based solar cells, which can be easily solution processed, are cheap and earth abundant, and offer essentially limitless opportunities for tuning the optical and electronic properties via molecular engineering.^1,2^ However, the efficiency of these devices has so far been low compared with inorganic solar cells. While photo-excitation of silicon and other inorganic semiconductors generates free charges, the low dielectric constant of organic semiconductors means that bound, localized Frenkel excitons with binding energies of ∼0.2−1 eV are typically generated in these materials.^3,4^ Dissociation of the exciton into free charges requires the exciton binding energy to be overcome. Designing a device that can perform this efficiently and also optimize the various other factors influencing power conversion efficiency is challenging. Therefore, understanding the detailed mechanisms of exciton formation, charge separation, and charge transport in these complex materials is crucial for optimizing the efficiency of OPVs.

Time-resolved x-ray spectroscopy can provide unique insight into time-dependent changes in electronic structure driving these processes. The core-level transitions accessed in the x-ray regime are localized at a particular atomic site in the system. This means that the x-ray absorption spectrum is sensitive to the local electronic structure in the vicinity of the absorbing atom. Since the elemental absorption edges can be separated by hundreds of electron volts or more, distinct elemental constituents of a sample can be easily separated, meaning that x-ray spectroscopies are an element-specific probe of the (time evolving) electronic structure. The soft x-ray (SXR) spectral range is of particular interest for organic systems, because this covers the K shells of carbon, nitrogen, and oxygen as well as the L edges of many heavier elements.

Over the past 20 years, there has been rapid development of time-resolved x-ray spectroscopy at both facility scale sources, such as x-ray free electron lasers and synchrotrons, and with table-top x-ray sources based on high-order harmonic generation (HHG). Synchrotrons provide x-ray pulses with 10s of picosecond duration at megahertz repetition rate and can also deliver femtosecond pulses at reduced repetition rates.^5^ Probing in the hard x-ray spectral region, these x-ray sources have been applied to time-resolved x-ray absorption spectroscopy (TR-XAS) of numerous systems relevant to solar energy conversion, including transition metal complexes used as photosensitizers,^6–8^ dye sensitized nanoparticles in solution,^9–11^ and perovskite nanocrystals in solution.^12^ The significantly shorter hard x-ray pulses available at x-ray free electron laser (XFEL) can push the timing resolution to the 10s to 100 fs level, with significantly increased pulse energy. This has allowed access to ultrafast spin crossover and wavepacket dynamics in transition metal complexes,^13–15^ ultrafast electron transfer in bi-metallic complexes^16^ relevant to photocatalysis, and hole dynamics in TiO_2._^17,18^ In addition to improving the temporal resolution in hard x-ray absorption spectroscopy, the high peak intensity of these pulses can reduce the effect of sample damage and enable “photon hungry” x-ray spectroscopic techniques such as valence to core x-ray emission and time-resolved resonant inelastic x-ray scattering (RIXS).^19–21^ There are a number of excellent reviews on application of time-resolved hard x-ray spectroscopy using these sources.^22–25^

As discussed above, access to the soft x-ray spectral range is necessary for the study of the organic semiconductors used in OPV devices. At the K edges of C, N and O, x-ray absorption excited dipole allowed 1s to 2p transitions, where the 1s orbital is strongly localized on the absorbing atom. Therefore, x-ray absorption spectroscopy (XAS) at these edges accesses the 2p contribution of the element to the valence electronic structure. Probing at the nitrogen and oxygen K edges, time-resolved soft x-ray absorption spectroscopy using synchrotron and XFEL sources has yielded new insights into transition metal complexes in solution,^26–31^ inorganic semiconductors in the solid state,^32,33^ chemisorbed molecules,^27^ and the photophysics of gas phase organic molecules.^34^ Relevant to this article, x-ray absorption spectroscopy at the carbon K edge with picosecond resolution has been used to observe x-ray spectroscopic signatures of a long lived triplet state in a pentacene film.^35^ Complementary to soft x-ray absorption, soft x-ray photoelectron spectroscopy (XPS) can probe charge transfer dynamics at surfaces or interfaces in photo-conversion systems with element specificity.^36^ Core-level XPS measures the binding energy of core-level electrons, which is sensitive to the charge in the vicinity of the absorbing atom through the chemical shift of the ionization potential. There has been significant progress on applying picosecond soft x-ray XPS to charge transfer across layered organic heterojunctions in the solid state,^37–39^ and the technique has more recently been extended to the femtosecond regime using XFEL sources.^40^

Recent advances in HHG-based x-ray sources and x-ray free electron lasers (XFELs) are enabling the generation of soft x-ray pulses with attosecond duration. Combining-order these sub-femtosecond x-ray pulses with an optical pump pulse, pioneering studies on molecular systems in the gas phase at the carbon and nitrogen edges using HHG-based sources have given new insights into the photophysics and photochemistry of gas phase molecules and molecular cations on ultrafast timescales.^41–46^ More recently, soft x-ray TR-XAS using HHG-based sources has been extended to liquid and solid state samples.^47–51^ A limitation of HHG-based x-ray sources is currently the low conversion efficiency and, therefore, the low flux in the SXR spectral region. This is overcome by recently developed attosecond XFEL sources,^52^ which provide dramatically increased pulse energies as compared to HHG-based sources. These sources have enabled demonstration of x-ray pump, x-ray probe absorption and photoelectron spectroscopy^53,54^ as well as some of the building blocks of nonlinear x-ray spectroscopy in the SXR.^55^

The exceptional timing resolution of these HHG-based and XFEL sources combined with the information content of soft x-ray absorption and photoemission spectroscopies promises to yield new insights into primary photo-induced processes in OPV materials. In this perspective, we highlight recent developments and future prospects for time-resolved soft x-ray spectroscopy of organic thin-film materials relevant to solar energy conversion. We focus on optical pump, x-ray probe measurements of organic materials in the solid state, which pose unique technical challenges due to low optical damage thresholds and poor thermal conductivity. The recent developments in HHG- and XFEL-based soft x-ray sources, which enable these measurements, are presented along with prospects for improving the photon flux of HHG-based sources and detection sensitivity. We discuss recent results at the carbon K edge, which have shown the promise of TR-XAS and XPS for tracking localization and charge transfer processes in these systems with atomic site specificity. A model for estimating the signal to noise ratio in time-resolved x-ray absorption measurements performed in transmission mode is developed. This is applied to examine feasibility of multi-edge x-ray spectroscopic measurements on organic semiconducting materials across the water window. Multi-edge time-resolved x-ray spectroscopy has recently been demonstrated in the extreme ultraviolet (XUV) spectral range on a layered heterojunction device,^56^ and in the thin-film materials WSe_2_^57^ and WS_2_^58^ at the O_3_ edge of W as well as the M_4,5_ and L_2,3_ edges of selenium and sulfur, respectively. Finally, we discuss methods for reducing and characterizing pump pulse-induced thermal heating and damage of these thin-film materials.

## II. ULTRAFAST DYNAMICS IN ORGANIC PHOTOVOLTAIC DEVICES

Organic photovoltaics have attracted a great deal of interest due to a number of potential advantages over conventional semiconductor solar cells: High optical absorption coefficients and the ability to be solution processed enables flexible, thin-film solar cells with low manufacturing costs.^59^ A key realization in the development of efficient organic photovoltaic devices is that dissociation of Frenkel excitons can be achieved efficiently at the interface of two organic materials (denoted donor and acceptor) with appropriate absolute HOMO and LUMO energies. This is because the energy gained by transferring the electron from the LUMO of the donor to the LUMO of the acceptor, and vice versa for the hole, can be sufficient to overcome the binding energy of the exciton. The first demonstration of this concept was in bilayer devices,^60,61^ consisting of a layer of donor material and a layer of acceptor material. The efficiency of these devices were limited by the exciton diffusion length of the donor material, which is typically ∼10 nm in organic materials.^62^ A technical breakthrough therefore came when Yu *et al.* introduced the bulk heterojunction (BHJ).^63^ This consists of an amorphous blend of the donor and acceptor materials, which increases the interfacial area between the donor and the acceptor materials, leading to a significant efficiency improvement in comparison with bilayer devices. Early research on BHJ devices focused on the mixture of P3HT and the fullerene derivative [6,6]-phenyl C_61_ butyric acid methyl ester (PCBM)^64,65^ in a thin film. By optimizing the morphology and crystallinity of these devices, efficiencies of close to 5% have been achieved^66^ in P3HT-PCBM BHJ solar cells. Optimization of the donor polymer materials to improve the light absorbing and charge transport properties led to an efficiency of ∼11% being achieved in a polymer-fullerene device.^67^ More recently, the efficiency of OPV devices has been significantly improved by the introduction of novel non-fullerene acceptor (NFA) materials such as Y6, with the polymer-Y6 blend PM6-Y6 achieving power conversion efficiency over 16%.^68,69^ In comparison to fullerene acceptors, NFAs have the advantage of tunable optical and electronic properties and stronger absorption. Y6 can also function as a single-component organic solar cell due to the intrinsic donor–acceptor structure of the molecule.^70^

There are many factors that contribute to the efficiency of a BHJ device. Both the chemical structures of the donor and acceptor and the morphology of the thin film play important roles in determining the optical and electronic properties of the device. One important aspect is improving our understanding of the fundamental exciton generation, transport, and separation dynamics, which are crucial in determining the overall efficiency. In a bulk heterojunction solar cell, there are a number of excited-state processes that lead to, and compete with, charge separation at the donor–acceptor interface. In a simplified picture, photo-absorption leads to the generation of excitons in the donor or acceptor material, which transport to the interface between the donor and acceptor forming a bound charge transfer state, and then finally a charge separated state. This competes with recombination processes for each excited state and the generation of other excited-state species, which limit the free charge yield.^62^ Time-resolved optical studies on BHJ devices have shown that the formation of charge transfer states at the donor–acceptor interface occurs on sub 100 fs timecales.^71^ Open questions remain in the role of vibrational coherence and charge delocalization in promoting charge separation in BHJ devices,^72–75^ neat polymers,^76^ and Y6 and other Y series acceptor molecules.^69^

## III. TIME-RESOLVED SOFT X-RAY SPECTROSCOPY

Time-resolved optical studies have been crucial for understanding many of the fundamental steps of charge photogeneration in OPV devices. However, the complexity of these systems and the presence of multiple excited-state species with overlapping optical spectra can make unambiguous assignment of species in optical studies challenging. Time-resolved x-ray spectroscopy offers a complementary method for probing excited-state processes with a number of distinct advantages over optical spectroscopy. The first is element specificity. The x-ray absorption energies of the K edge of light elements are separated by more than 100 eV, and the absorption and emission from the K shell of these elements probe the local electronic structure in the vicinity of the atomic site. In a simplified picture, the pre-edge absorption fine structure (NEXAFS) at the K edge is sensitive to the atomic 2p orbital contribution of a given element to the valence orbitals of the system. Viewing the dynamics from multiple elemental absorption edges can therefore reduce the spectral congestion and allows for a simplified understanding of the electronic structure of the excited-state species in terms of molecular orbitals.

In addition, the binding energy of a core level electron is sensitive to the oxidation state of the corresponding atom.^77^ This leads to chemical shifts in absorption or photoemission features, which are therefore sensitive to the electron or hole density at a given element. Where an increase in the oxidation state of the element leads to an increase in the core-level binding energy and vice versa. A change in binding energy can be detected via photoemission or as a shift in the x-ray absorption features. The shift will depend on the chemical environment of the atom and allows for tracking of electron and hole density through a complex system with multi-edge x-ray spectroscopic measurements. This technique has been demonstrated in TR-XAS of inorganic semiconductors, nanoparticles, mononuclear transition metal complexes, and bimetallic complexes.^12,15,32,78–80^ Section III A discusses ongoing developments in ultrafast soft x-ray sources, which are beginning to enable these methodologies in more technically challenging thin-film materials in the soft x-ray range.

### A. Ultrafast soft x-ray sources

The experimental realization of time-resolved soft x-ray spectroscopy on OPV materials requires ultrafast x-ray sources with a number of important properties such as the following:

• The x-ray pulse duration and timing uncertainty between the x-ray probe and pump pulse should be shorter/better than the timescale dynamical process of interest.
• The x-ray photon energy should be sufficient to access the relevant absorption edges for the studied sample. A broadband x-ray source in conjunction with a spectrometer to disperse the x-rays following interaction with the sample is advantageous since it allows the full structure around the edge to be resolved in a single shot, otherwise tuning of the central photon energy is required.
• The photon flux should be sufficient to resolve the small pump-induced changes in absorbance (calculations of the required count rate for realistic experimental conditions are presented in Sec. IV A).

Synchrotrons provide a stable, high repetition rate and high flux x-ray source with widely tunable photon energy up to the hard x-ray region. However, the pulse duration is long ( ∼100 ps), which limits its applicability to ultrafast dynamics. Through time slicing, the pulse duration can be reduced somewhat to ∼100 fs, but this limits the repetition rate and flux of the source.^5^ X-ray free electron lasers (XFELs) provide very high pulse energy (up to the mJ level) and short pulse duration down to the attosecond level^52^ (although in self-amplified spontaneous emission mode, which is typical, the pulse duration is closer to 10 fs). Current XFEL sources such as the Linac Coherent Light Source (LCLS),^81^ the Spring-8 Angstrom Compact Free Electron Laser (SACLA),^82^ the Pohang Accelerator Laboratory X-ray Free Electron Laser (PAL-XFEL),^83^ SwissFEL,^84^ and FERMI^85^ produce x-ray pulses at ∼100 Hz repetition rate. The photon energy of these sources is tunable with different facilities accessing different photon energy ranges from the XUV to the hard x-ray. The European XFEL^86,87^ has increased this repetition rate up to 27 kHz covering photon energies 250–25 keV, and the FLASH facility currently operates up to 8 kHz in the XUV to SXR spectral range.^88,89^ Future XFEL facilities will push the repetition rate further,^90^ with LCLS-II planning to operate up to 1 MHz repetition rate. The high pulse energies and short pulse duration make XFELs a unique x-ray source and open up the possibility of multidimensional nonlinear x-ray spectroscopy.^91^ One disadvantage of XFELs for pump probe measurements is that they are inherently unstable, and synchronization with an optical pump pulse is challenging,^92^ leading to significant timing jitter of tens of femtoseconds. The temporal resolution in a pump probe measurement is therefore limited by the accuracy that the arrival time can be measured on a shot to shot basis.^93,94^ Improvements in synchronization and the accuracy of the arrival time measurements are required to achieve the few-femtosecond temporal resolution necessary for understanding the primary exciton dynamics.

#### 1. SXR HHG

High harmonic generation has been shown to be a promising source of x-rays for time-resolved x-ray absorption spectroscopy. The process inherently produces short (<1 fs), extremely broadband x-ray pulses, which are phase locked to a driving laser field. X ray photon energies up to 1.5 keV from HHG have been reported,^95^ and the bandwidth of the radiation extends from this energy to that of the driving laser. This means that these sources can be used to measure XAFS spectra at multiple edges in a single shot without the need to scan the photon energy. They can also be easily incorporated into stable interferometers for optical pump, x-ray probe measurements with attosecond temporal resolution. However, the major challenge for these sources is their low conversion efficiency, which falls off steeply with increasing photon energy. This low soft x-ray flux cannot easily be solved as the generation processes of HHG in a noble gas is governed by the interlinked dynamics of ionization clamping at high intensities, pulse reshaping, and phase matching with the generated SXR. When driven by Ti:sapphire laser systems at 800 nm, the maximum photon energy that can be reached with useful flux levels is ∼200 eV. By increasing the wavelength of the drive laser field beyond the 800 nm, the photon energy of the harmonics can be extended from the XUV spectral range into the water window. This exploits the $\lambda^{2}$ scaling of the HHG cutoff with drive laser wavelength. Novel gas cell designs, modeling of the propagation, and potentially large area driving beams will be required to substantially increase the SXR flux. The technology and phase-matching considerations for SXR HHG have been reviewed in detail in Refs. 96 and 97.

**FIG. 1.**
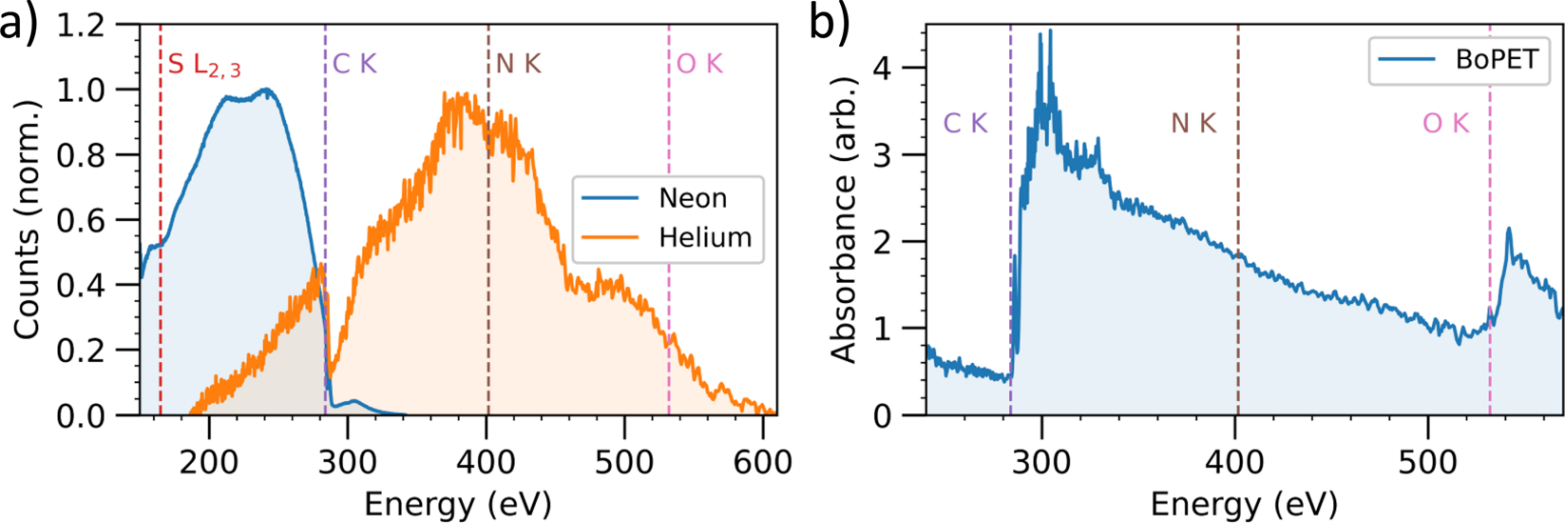
Soft x-ray high harmonic generation and its application to x-ray absorption spectroscopy across the water window. (a) HHG spectra generated in neon and helium, driven by 1.8 *μ*m few-cycle pulses. Absorption from the carbon K edge at ∼285 eV is apparent due to carbon contamination of the x-ray optics. (b) Application of the source to x-ray absorption spectroscopy of a biaxially oriented polyethylene terephthalate (BoPET) film at the carbon and oxygen edges.

First-generation SXR HHG sources utilized a Ti:sapphire pumped optical parametric amplifier (OPA) to increase the wavelength of the drive laser field. This gives signal and idler pulses between ∼1.1−1.6 and ∼1.6−2.0 *μ*m, respectively. Post-compression of the ∼30 fs signal or idler pulses from the OPA to the few-cycle duration is performed either in a hollow core fiber^98–101^ or via filamentation in an extended gas cell.^102^ High-order harmonics are generated in a high pressure gas cell, filled with either helium or neon. The phase-matching pressure scales with $\geq \lambda^{2}$, and multiple atmospheres of pressure are required for phase matching SXR HHG in helium driven at 1.8 *μ*m.^97,103^ The maximum photon energy reached depends on the drive wavelength and the generation medium. Driving HHG in helium at 1.4 *μ*m is sufficient to achieve photon energies just beyond the carbon K edge.^99^ Employing the idler of a 800 nm pumped OPA (typically 1.8 *μ*m) is sufficient to generate HHG across the water window extending to the oxygen K edge. Figure 1 shows example HHG spectra recorded in our lab. HHG in either neon or helium was driven by few-cycle pulses at 1.8 *μ*m. HHG in neon covers photon energies up to ∼330 eV, spanning the carbon K edge and the L edges of chlorine and sulfur. HHG in helium spans the K edges of carbon, nitrogen, and oxygen. The photon flux of the source at generation is approximately $3\times 10^{6}$ ph/s/1%BW at the carbon K edge and drops to $5\times 10^{4}$ ph/s/1%BW and $1\times 10^{3}$ ph/s/1%BW at the nitrogen and oxygen edges, respectively. This photon flux is sufficient to perform XAS at the oxygen K edge in ∼ hours^101^ and at the carbon K edge in ∼ minutes.^104^ This is demonstrated in Fig. 1 with a XAS measurement of a biaxially oriented polyethylene terephthalate (BoPET) film across the water window from the carbon to the oxygen edge. However, as discussed quantitatively in Sec. IV A, time-resolved spectroscopy of OPV materials requires detection of much smaller absorption changes at a number of delay times. While the flux at the C K edge is sufficient for such measurements extending these to higher lying absorption edges will be extremely challenging with current sources. Therefore, in Secs. III A 2 and 3, we discuss possibilities for increasing the x-ray flux at generation through the use of high repetition rate laser sources and in increasing the efficiency of photon transport and detection.

#### 2. High repetition rate laser and XFEL sources for time-resolved x-ray spectroscopy

Improving the average power of HHG-based soft x-ray sources will require technological development to scale both the repetition rate and average power of the drive laser. Third-generation femtosecond laser sources based on optical parametric chirped pulse amplification (OPCPA) pumped by thin disk, Yb, lasers^105^ provide significant improvements in this regard as compared to Ti:sapphire lasers. These sources are also wavelength tunable and can provide few-cycle pulse duration. Another promising avenue for increasing the average power of the drive laser field is direct post-compression of laser systems operating at longer wavelength in hollow core fibers. For example, Yb (1.03 *μ*m) or Tm (2.05 *μ*m) fiber lasers,^106–110^ or CPA systems at > 2 *μ*m using doped Zn:Se and other novel gain media.^111^ There have been a number of promising results using some of these sources to improve both the flux and photon energy range of SXR HHG. For example, OPCPA laser technology has already been used to generate harmonics spanning the water window at 100 kHz^112^ and to reach photon energies of 1.6 keV with flux sufficient for XAS at the Fe L edge (700 eV).^95^

For reasons of the pump intensity and fluence-induced effects (discussed herein), good signal to noise in TR-XAS measurements at XFELs also benefits from high repetition rate for the optical pump laser and the SXR probe source. The first generation of XFELs based on normal conducting accelerators operating at repetition rates of ∼100 Hz have thus far proved unsuitable, especially as the inherent high power of the x-ray pulses must be strongly attenuated to avoid probe-induced effects. High repetition rate XFELs with superconducting linacs overcome this constraint. This is demonstrated by a recent work^40^ (discussed later) at the FLASH facility where 4000 pulses per second were available. Likewise, other machines now becoming available (e.g., LCLS II) moving toward 1 MHz repetition rate will prove suitable. Improvements in synchronization with external lasers through better intrinsic stabilization, improved time tools and seeding are likely to permit < 10 fs temporal resolutions in future measurements. Operating modes such as enhanced SASE that generate attosecond near transform limited bandwidth (of 5–10 eV) may be sufficient to capture the relevant NEXAFS spectral region with single shot acquisition as is possible with HHG sources.

#### 3. Efficient x-ray coupling and detection

Another promising route to improving the signal to noise in soft x-ray absorption spectroscopy is in optimizing the efficiency of soft x-ray transport optics and spectrometers. The most widely adopted SXR spectrometer design uses a variable line spaced diffraction grating in combination with either a MCP or x-ray CCD camera for photon detection (termed VLS spectrometer). These gratings typically have first-order diffraction efficiencies less than 1% at photon energies above ∼300 eV. X ray CCD cameras have excellent linearity and quantum efficiency close to 1 in the SXR. However, the readout time for these detectors in low noise configurations is slow. Fast readout of the CCD chip (which is often required in TR-XAS applications) incurs a significant increase in the readout noise, making this the dominant noise source.

The throughput of SXR spectrometers can be improved by optimizing the diffraction efficiency of the dispersive element. Transmission gratings can achieve first-order diffraction efficiency of ∼10%;^113^ however, this is typically at the expense of spectral resolution. Reflective and transmissive zone plate gratings also offer significantly improved efficiency as compared to plane diffraction gratings.^114,115^ Using a reflective zoneplate spectrometer, Kleine *et al.*^114^ have demonstrated a factor of ∼17 improvement in throughput as compared to VLS-spectrometers at the C K edge and a factor of ∼30 at the N K edge with an energy resolution ( ΔE/E of 860) across the water window.

Continued developments in soft x-ray cameras will also significantly improve the performance of these spectrometers. The incorporation of CMOS technology in hybrid detectors is giving significant improvement in the readout rate and noise level of cameras in the x-ray range.^116,117^ The noise performance of recently developed CMOS detectors is sufficient for single photon counting in the soft x-ray.^118^ Photon counting greatly reduces the noise in x-ray detection, but requires that the number of photons per pixel in each shot is low. Single-shot measurement of the spectrum is therefore optimal for photon counting and can also reduce the influence of other noise sources, such as instabilities in the x-ray source. Recently developed CMOS detectors for the European XFEL combine low noise levels with MHz repetition rate readout; therefore, making them the ideal detector for time-resolved spectroscopy in the SXR.^119^

### B. Time-resolved soft x-ray spectroscopy of OPV materials

The developments of soft x-ray HHG-based sources and high repetition rate XFEL sources are already enabling time-resolved x-ray spectroscopic studies of OPV materials at the carbon K edge with femtosecond timing resolution. In Sec. III B, we review two recently published results^40,47^ demonstrating the utility of C K edge spectroscopy to probe charge localization in an organic semiconducting polymer and charge transfer dynamics across a bilayer heterojunction device.

#### 1. Ultrafast exciton localization in P3HT

In our recent work,^47^ we employed TR-XAS at the carbon K edge with 15 fs timing resolution to probe exciton dynamics in P3HT. As described in Sec. II, P3HT has played an important role in the development of BHJ solar cells, and P3HT:PCBM BHJ solar cells were the first BHJ devices to achieve relatively high efficiencies. In P3HT thin films, crystalline regions of lamellae stacked polymer chains are thought to promote delocalization of the exciton between polymer chains. When incorporated into a BHJ devices, this increases the efficiency of charge separation by promoting ultrafast coherent charge transfer to the donor–acceptor interface. The charge transfer dynamics of the delocalized exciton compete with localization processes driven by coupling of the electronic wavefunction to the vibrational and torsional modes of the polymer. Previous work on P3HT had suggested that the localization of the exciton occurs on a sub-100 fs timescale;^120,121^ however, probing these localization processes directly is challenging for existing ultrafast optical techniques both due to the lack of direct sensitivity to the spatial extent of the excited-state wavefunction and the extremely short timescales often involved.

**FIG. 2.**
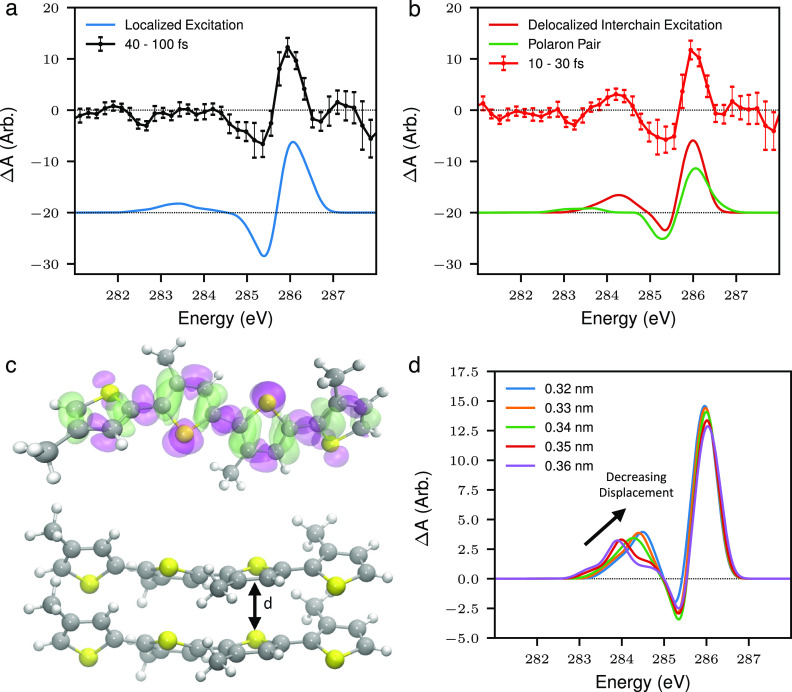
TR-XAS of P3HT at the carbon K edge. (a) and (b) The differential absorption spectrum of P3HT at short (10–30 fs) or long (40–100 fs) time delays following photo-excitation of the $\pi \to \pi^{*}$ transition with a 15 fs optical pulse and a comparison with TDDFT simulations modeling a localized exciton, a polaron pair and a delocalized exciton. (c) Electron density difference from the TDDFT simulation of an isolated tetrathiophene oligomer (modeling localized exciton), where purple corresponds to a loss of electron density, and green corresponds to a gain. (d) Calculated TR-XAS spectrum for a tetrathiophene dimer as a function of *π* stacking distance. Figure reproduced with permission from Garratt *et al.*, Nat. Commun. **13**, 3414 (2022). Copyright 2022 Authors, licensed under a Creative Commons Attribution (CC BY) license.

As discussed earlier, x-ray spectroscopy is uniquely sensitive to electronic structure. This allows for exciton localization to be probed at the carbon K edge via its effect on the local electron density at the carbon atoms. Figure 2 shows transient XAS spectra of P3HT pumped by a 15 fs optical pulse at the carbon K edge at short time delays [10–30 fs, Fig. 2(b)] and longer time delays [40–100 fs, Fig. 2(a)]. There are two main spectroscopic features in the XAS spectrum with differing timescales. The strongest effect is a blue shift in the C1s $\to \pi^{*}$ transition, giving rise to the differential profile at around 286 eV, which persists for the 100 fs time delay range addressed in the measurement. The second is a transient increase in absorption at around 285 eV at short time delays (16 ± 8 fs time constant from an exponential fit).

In P3HT, photo-excitation of the $\pi \to \pi^{*}$ transition transfers electron density away from the conjugated carbon atoms at the center of the thiophene ring toward the sulfur as shown in the electron density difference map [Fig. 2(c)]. This is a consequence of the underlying molecular orbital structure of the thiophene monomer, with the HOMO being an aromatic orbital with little electron density on the sulfur and the LUMO having a quinoid structure, strongly coupled to the sulfur 2p orbitals. This transfer of electron density leads to the long lived blue shift in the C1s $\to \pi^{*}$ transition. This effect is reproduced in time-dependent density functional theory (TDDFT) simulations of a tetrathiophene oligomer in its lowest lying excited state. As shown in Fig. 2(a), the transient XAS spectrum of this species reproduces the experimental signal at 40–100 fs time delay.

The short lived, transient feature, on the other hand, appears below the 1s $\to \pi^{*}$ transition, indicating that it is due to an increase in electron density at the carbon atoms. Based on the timescale of the feature and previous work on P3HT, it could be either due to polaron pair formation or inter-chain exciton delocalization. The latter was modeled with TDDFT simulations by considering the lowest lying excited state of *π* stacked tetrathiophene oligomers. The *π* stacking leads to a delocalization of the wavefunction between oligomer chains. The net effect of which is to increase the electron density at C 2p orbitals, which in turn leads to a red shift-induced absorption at around 285 eV [see Fig. 2(b)]. The amplitude and energy of this feature depends on the *π* stacking distance with smaller distances, leading to a blue shift and increase in amplitude of the feature. Modeling of the contribution of polaron pairs to the x-ray spectrum using the TDDFT approach did not reproduce an increase in absorption observed at short time delays. Based on these assignments, the experimental data indicate that photo-excitation of P3HT generates excitons that are delocalized between polymer chains, which then rapidly localize within 20 fs to form either localized excitons or polaron pairs. Further developments in the experimental resolution and noise level as well as in the theoretical modeling of the time-dependent x-ray absorption spectrum in these systems^122–125^ will enable access to the details of what drives this localization.

#### 2. Charge transfer in a bilayer heterojunction with core-level XPS

A complementary technique to TR-XAS for probing ultrafast dynamics in OPV materials is time-resolved core-level XPS. TR-XPS measures the binding energy of core-level photo-electrons, which is in turn sensitive to the charge density at the atom from which the electron is emitted. As with TR-XAS, this atomic site-specific probing of charge density is in principle a powerful technique for tracking ultrafast processes in OPV materials. This is demonstrated in work by Roth *et al.* who employ time-resolved carbon K edge XPS to track electron transfer in a copper-phthalocyanine (CuPc)-C60 bi-layer heterojunction.^37,38,40^ Photo-excitation of the CuPc chromophore leads to an electron transfer from the CuPc donor to the C60 acceptor, which manifests as a blue shift in the kinetic energy of the C1s photo-line associated with the C60 layer due to increased screening of the core-hole by these electrons.^38^

**FIG. 3.**
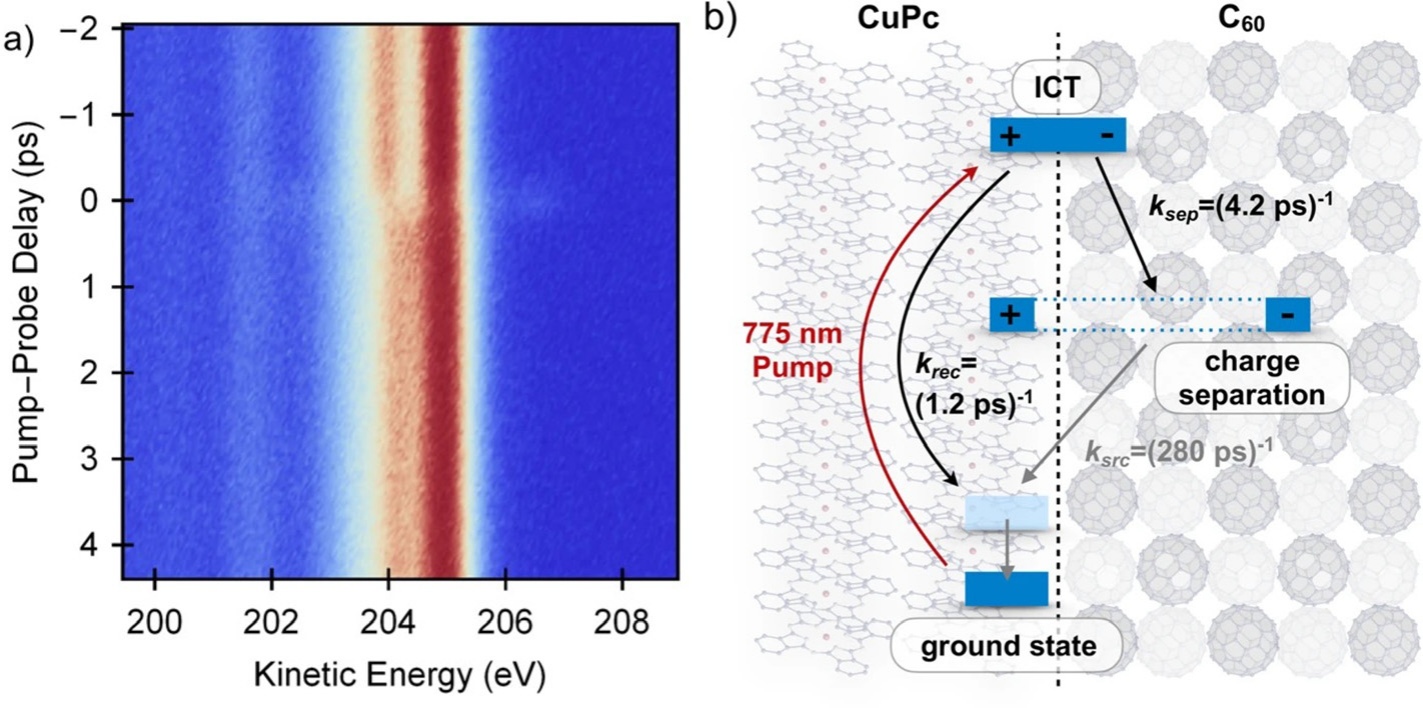
Time-resolved XPS of a carbon K edge XPS of a layered CuPc-C60 heterojunction at the carbon K edge performed at FLASH. (a) Time-dependent XPS over the first 5 ps following photo-excitation of the CuPc donor at 775 nm. (b) Illustration of a kinetic model describing the experimental data. Figure adapted with permission from Friedrich Roth *et al.*, Nat. Commun. **12**, 1196 (2021). Copyright 2021 Authors, licensed under a Creative Commons Attribution (CC BY) license.

TR-XPS measurements on femtosecond timescales performed at FLASH [shown in Fig. 3(a)] observed an instrument response limited appearance of a blue-shifted photoemission line [referred to as C60(1)] due to electron transfer to the C60 layer. This is assigned to direct excitation of interfacial charge transfer (ICT) states by the 775 nm pump pulse. Afterward, a bi-exponential decay in the amplitude of the C60(1) feature is observed. As illustrated in Fig. 3(b), the two decay components are associated with electron–hole recombination (time constant 1.2 ± 0.3 ps) or dissociation of the ICT into separated charges with a time constant of 4.2 ± 0.8 ps leading to the residual blue shift in the C60 absorption at long time delays. The timescale for decay of the ICT state obtained from the TR-XPS signal is consistent with previous optical studies on this system.^126,127^ However, the optical studies were not sensitive to the long lived charge separated product formed from this state, highlighting the advantage of x-ray spectroscopy for identifying charge photo-generation pathways in complex, multi-component devices.

## IV. FUTURE PROSPECTS

The aforementioned two examples employed time-resolved x-ray spectroscopy at a single elemental absorption edge to resolve localization and charge transfer processes in OPV materials. The true power of x-ray measurements on these systems will likely be in multi-edge spectroscopic measurements, which enable tracking of the movement of electrons and holes between different sites in the material or device. In Secs. IV A and IV B, we review the feasibility of such measurements based on the current and future performance of HHG-based x-ray sources. We also discuss the challenges posed by sample damage and heating, which must be tackled in future studies.

**FIG. 4.**
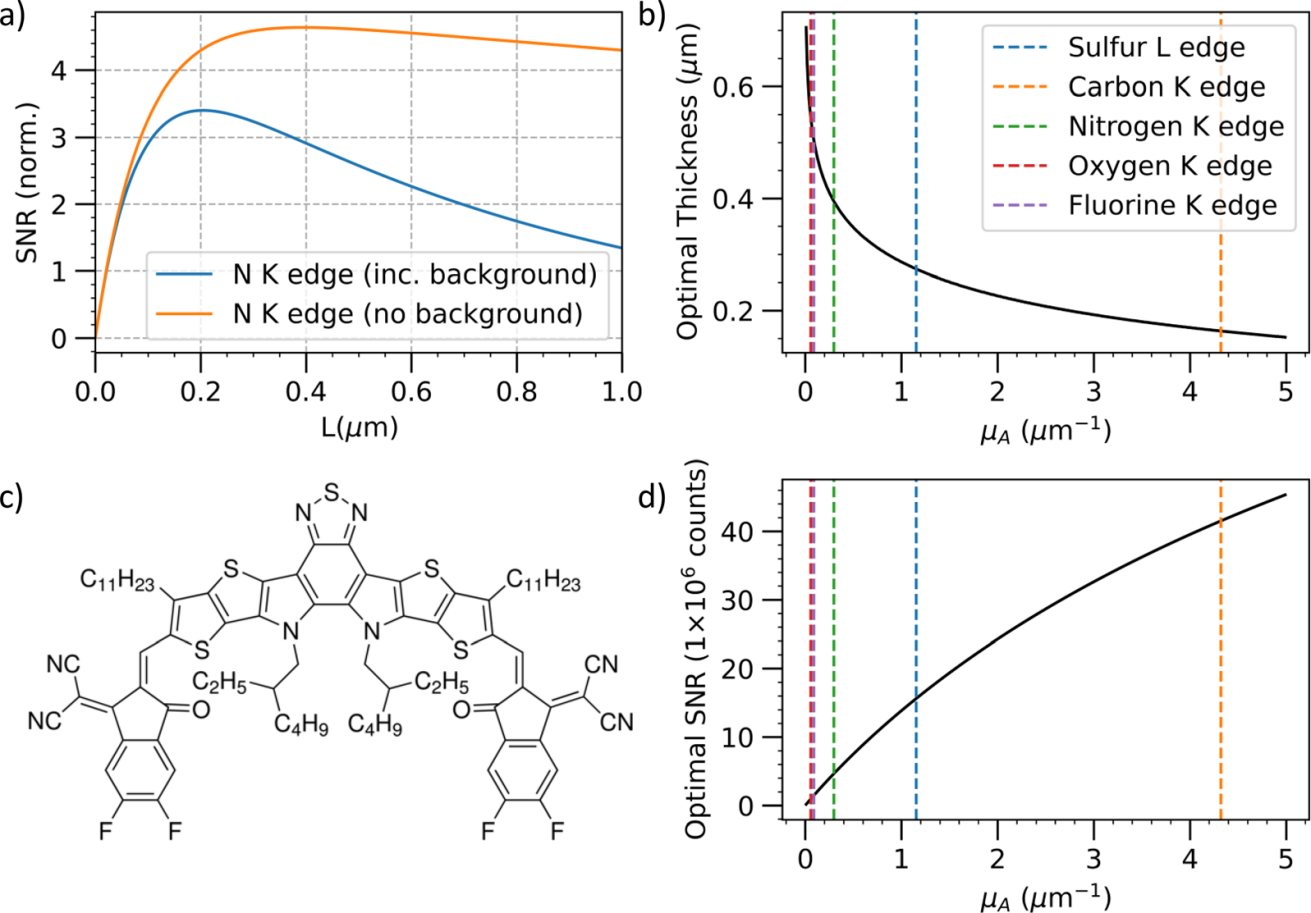
Modeling the signal to noise ratio for an optical pump, soft x-ray probe experiment on the novel small-molecule acceptor Y6. Calculations assume an optical absorption coefficient of 10.9 *μ*m^– 1^ and a pump pulse fluence of 0.3 mJ/cm^2^. X-ray absorption coefficients at each edge are summarized in Table I. (a) Signal to noise ratio at the N K edge as a function of sample thickness. The blue line includes the effect of background absorption, while the orange line neglects this contribution. (b) Optimal sample thickness as a function of x-ray absorption coefficient, *μ_A_* assuming $\mu_{B}=0$. (c) The chemical structure of Y6. (d) Signal to noise at the optimal sample thickness as a function of x-ray absorbance.

A promising system for future measurements is the non-fullerene acceptor molecule Y6 (and related materials). Y6 has a molecular structure composed of alternating acceptor and donor moieties in the form A-DAD-A. It exhibits strong absorption in the visible-NIR spectral range (650–900 nm), and recently, a power conversion efficiency approaching 18% was reported.^128^ The acceptor moiety in Y6 is a benzothiadiazole (BT) group, which is electron-deficient (see Fig. 4 Ac). Comprising 2 N atoms and 1 S atom at this active acceptor site makes it very amenable to x-ray spectroscopy, which is highly sensitive to local changes in electron density at the probing site. In this case, the probing can be at the S L edge (165 eV), S K edge (2475 eV), or the N K edge (410 eV) with S L, C K, and N K edge accessible in principle to HHG-based sources. The flanking acceptor groups have high absorption and incorporate F atoms where the electron dynamics can also be probed (F K edge 697 eV). All edges in the system are accessible to the emerging family of high repetition rate XFELs at European XFEL, FLASH (DESY), and LCLS II, making this a promising candidate for fully characterizing the electron dynamics by integrating time-resolved x-ray spectroscopy observations at multiple sites. In Sec. IV A, we examine the feasibility of future measurements on Y6.

### A. Signal to noise considerations

Given the low flux of HHG-based x-ray sources in the soft x-ray range and the low optical damage threshold of OPV materials, it is crucial to optimize the signal to noise ratio (SNR) in the TR-XAS signal. Optical pump x-ray probe measurements on these materials are challenging from a SNR perspective due to the large difference in absorption cross section between the pump and probe. OPV materials have high optical absorption coefficients on the order of 10 *μ*m^–1^, while the x-ray absorption coefficient at a given elemental absorption edge in the SXR is typically a factor of 2–10 lower than this (depending on the exact structure of the molecule and the number of equivalent absorbing atoms). Therefore, the thickness of the thin film must be carefully chosen to balance the need for thicker samples to optimize the noise level in the x-ray absorption measurement and thinner samples to optimize the overall excitation fraction in the probed sample volume. OPV materials can be solution processed and spin coated onto a variety of substrates. There are therefore many ways to control the thickness of the films, such as varying the solution concentration, or the rotation speed of the spin coater.^104^

In what follows, we outline a simple model for predicting the optimal film thickness and achievable SNR in TR-XAS measurements. The model is an extension that is described in Ref. 129 for static spectroscopy to time-resolved experiments. We focus on XAS performed in a transmission geometry; however, a similar approach can be applied to total and partial fluorescent yield (TFY/PFY) detection at XFELs.

In a TR-XAS experiment, the quantity we are interested in measuring is the change of sample absorption induced by the pump pulse at a certain elemental absorption edge, $\Delta \mu_{D}$. This is determined by the fraction of photo-excited molecules, *η*, and the underlying x-ray absorption coefficient of these photo-excited species. As an approximation, we assume that this is equal to that of the ground state species, *μ_A_*, so that $\Delta \mu_{D}=\eta \mu_{A}$. This therefore approximates the magnitude of the pump-induced bleaching of the ground state absorption features. For measurements performed in transmission, this small change in absorption due to optical pumping must be resolved on top of a large absorption background denoted $\mu_{T}=\mu_{A}+\mu_{B}$, which has contributions both from the element whose edge is addressed with the x-ray probe (*μ_A_*) and elemental constituents of the material or device with lower lying absorption edges (*μ_B_*).

The fraction of excited molecules depends on the sample thickness, optical absorption coefficient, *μ*_vis_, and the pump pulse fluence, *F*_0_, as

$$\eta =\frac{F_{0}(1-e^{-\mu_{\text{vis}}L})}{nLE_{\text{photon}}},$$ (1)

where n is the number density of the sample, and $E_{\text{photon}}=hc/\lambda$. The fraction of excited molecules is therefore maximized for thin samples and follows an approximately 1/L relationship with sample thickness.

To isolate the pump-induced change in absorption, the transmitted x-ray intensity is measured with and without the pump pulse (denoted *I_p_* and *I_up_*, respectively), and the total measured signal is proportional to the ratio $I_{p}/I_{up}$. The contribution of the underlying change in absorption to this can be approximated as

$$S\approx \eta \mu_{A}L.$$ (2)

Assuming shot noise limited x-ray detection, the noise in the measured signal is given by

$$N=\frac{I_{p}}{I_{up}}{\left(\frac{1}{I_{up}}+\frac{1}{I_{p}}\right)}^{\frac{1}{2}}.$$ (3)

If we assume that the change in absorption due to pumping of the sample is small in comparison to the total sample absorption (i.e., $I_{p}\approx I_{up}=I_{t}=I_{0}e^{-\mu_{T}L}$), then the signal to noise ratio simplifies to

$$\mathrm{SNR}\approx \eta \mu_{A}L\sqrt{\frac{I_{t}}{2}}.$$ (4)

Figure 4 summarizes application of this model to Y6. The optical absorption coefficient of Y6 at around 800 nm is^130^ 10.9 *μ*m^– 1^. It also has multiple elemental x-ray absorption edges C, N, O, F, and S accessible with either HHG or XFEL sources. Each of these edges has different x-ray absorption cross sections and levels of background absorption due to lower lying atomic constituents. Figure 4(a) shows the dependence of SNR on film thickness at the nitrogen K edge both with and without the contribution of background absorption from carbon and sulfur atoms. There is an optimum sample thickness, where the SNR at this edge reaches ∼3 for 10^6^ detected photons. For thicknesses below this optimum, the excitation fraction is increased, but the sample is not thick enough to achieve good signal to noise in the x-ray absorption measurement. The decrease in SNR for thicker sample is due to the reduction in excitation fraction as the thickness increases. Neglecting background absorption increases the optimal sample thickness and the optimal SNR, which can be obtained. This is because the background absorption reduces the transmitted intensity, therefore increasing the noise. In general, the edges with weaker absorption due to fewer atoms or underlying cross section require thicker samples to optimize the SNR [Fig. 4(b)]. In addition, the optimal SNR, which can be achieved for the same detected count rate and pump pulse fluence, is reduced, and the highest SNR is achieved at the C K edge, which also has the largest x-ray absorption coefficient [Fig. 4(c)].

The total number of detected counts required to achieve a SNR of 10 at each absorption edge is summarized in Table I. For this estimate, we have assumed a pump pulse fluence of 300 *μ*J/cm^2^. At the carbon K edge, approximately 10^5^ total detected photons are required for a SNR of 10. With current HHG-based soft x-ray sources delivering a photon flux of $∼10^{6}$ photons/s/ 1% BW at 300 eV, and assuming a transport/detection efficiency of 0.1%, a detected count rate of approximately 10^2^ cps can be expected in a 0.3 eV window at 300 eV. Note that an energy resolution of 1000 or more is required to resolve the NEXAFS features at the K edges of light elements, so we are using the HHG flux in 0.1% bandwidth for these estimates. A total integration time of ∼10 minutes (per delay point) is therefore required for experiments at the C K edge with current sources. With improvements to the soft x-ray flux and detection efficiency discussed in Sec. III A 3, this can likely be reduced by two orders of magnitude in future, enabling systematic studies of multiple samples to be performed.

**TABLE I.** Summary of the relevant parameters for estimating the signal to noise in TR-XAS measurements on Y6, which has an optical absorption coefficient of 10.9 *μ*m^–1^. *μ_A_* and *μ_B_* are the pre- and post-edge x-ray absorption coefficients obtained from the CXRO database.^131^ L_opt_ is the optimal film thickness in micrometers at each edge, estimated from Eqs. (2) and (3) including the effect of background absorption. The count rate required to achieve a SNR of 10 assumes a pump fluence of 300 *μ*J/cm^2^.

	*μ_A_* (*μ*m^–1^)	*μ_B_* (*μ*m^–1^)	L_opt_ (*μ*m)	Counts for SNR = 10
S L edge	1.2	1.3	0.21	$6\times 10^{5}$
C K edge	4.3	0.9	0.15	$7\times 10^{4}$
N K edge	0.3	2.3	0.20	$9\times 10^{6}$
O K edge	0.1	1.3	0.26	$2\times 10^{8}$
F K edge	0.1	0.7	0.30	$6\times 10^{7}$

Compared to the C K edge, the photon flux of HHG sources is reduced by approximately two or three orders of magnitude at the N and O K edges, respectively.^101^ Therefore, HHG-based time-resolved studies at these absorption edges on OPV materials will likely only be feasible with improvements to detection efficiency and photon flux discussed in Sec. III A 3. XFELs are therefore a more promising route to performing measurements at these edges in the near-term. Beamlines offering monochromatized x-ray pulses in the soft and tender x-ray range are available at a number of facilities around the world (LCLS, the European XFEL, FLASH, SwissFEL, and PAL-XFEL). At these beamlines, bulk sensitive time-resolved absorption measurements can be performed with fluorescence detection. The timing resolution is typically limited to 20–50 fs depending on the resolution of the monochromator. In partial fluorescence yield XAS, the fluorescence is spectrally resolved and integrated for a given element to give the absorption spectrum. This method has significant advantages over transmission or total fluorescence yield XAS for low concentration elemental absorption edges with large non-resonant backgrounds^132^ (as is the case for the N, O, and F edges in Y6).

### B. Sample damage and heating

The maximum attainable signal levels, which can be achieved in pump probe measurements on solid state systems, are often limited by sample damage. In the case of TR-XAS performed with HHG-based sources, sample damage by the x-ray pulse is negligible due to the extremely low pulse energies, and the main risk is from the optical pump pulse. Organic semiconductors are highly photosensitive, and the pump pulse fluence must be kept low (typically below 1 mJ/cm^2^) to avoid permanent sample damage. Even below the damage threshold, bi-molecular effects such as exciton–exciton annihilation^133^ will have a large impact on the measured lifetimes and at relatively low excitation densities. Taking P3HT as an example, optical studies^134^ suggest that to avoid exciton–exciton interactions, the pump pulse fluence has to be reduced to below ∼5 *μ*J/cm^2^. These low fluences represent a challenge, even for optical transient absorption spectroscopy; therefore, other approaches such as scanning the pump pulse fluence^135,136^ to characterize and subtract nonlinear component of the signal may be more suited to time-resolved x-ray spectroscopies.

In addition to sample damage and high intensity effects, an additional major challenge for future TR-XAS on OPV materials is thermal heating by the pump beam. Organic semiconductors and polymers typically have low thermal conductivity, and during a pump probe experiment, a large portion of the energy deposited by the pump pulse ultimately dissipates as heat in the sample. If this heat is not fully conducted away from the pumped volume between laser pulses, the average temperature of the sample can significantly increase. Due to the low penetration length of soft x-rays, experiments are performed in vacuum meaning that there is no convective cooling of the sample from the surface, which is a significant source of heat loss for optical experiments performed in air.

The temperature response of the sample T(t) after a single laser pulse is given by

$$T(t)=T_{0}e^{-\gamma t},$$ (5)

where $T_{0}=\frac{F(1-e^{\mu_{\text{vis}}L})}{C_{p}\rho L}$ is the initial temperature rise due to the pulse, which depends on the heat capacity, C_*p*_. *γ* describes the dissipation of heat in the sample and will depend on the thermal conductivity (*σ*) of the sample and the sample geometry. In general, accurate calculation of time and spatially dependent sample heating by a laser pulse requires numerical modeling of the three dimensional heat equation.^137,138^

Under continuous illumination by a pump pulse train frequency, f_*rep*_, the sample temperature equilibrates to

$$T_{ss}=\frac{F(1-e^{\mu_{\text{vis}}L})}{\rho {\text{LC}}_{p}(1-e^{-\gamma /f_{\text{rep}}})}.$$ (6)

Therefore, the equilibrium temperature depends strongly on the repetition rate of the laser and *γ*. If $\gamma \gg 1/f_{\text{rep}}$, the laser pulses will not induce a significant increase in the sample temperature. On the other hand, if *γ* is comparable to or less than $1/f_{\text{rep}}$, the heat deposited by the pulse train accumulates in the sample, increasing the sample temperature. In this case, the equilibrium temperature can be approximated by continuous wave (CW) heating of the sample by a laser of equivalent average power, which in turn can be estimated analytically or can be calculated numerically by solving the heat equation for the sample geometry (for example, including sample substrates).

In addition to elevating the equilibrium temperature of the sample, the thermal response of the sample to optical pumping can lead to time-dependent changes in the transient absorption spectrum. Optical studies on organic polymers have found that temperature modulations due to optical pumping can dominate the transient absorption signal on nanosecond timescales.^139,140^ In order to avoid these temperature-induced changes being misinterpreted as excited-state species, control measurements such as comparison of the transient absorption signal with the change in spectrum due to thermal heating are required to assign the thermal response. Similar care must be taken when interpreting long-lived signatures in x-ray absorption experiments on low thermal conductivity samples. Hayes *et al.*^141^ have found that both the optical and O K edge x-ray transient absorption signals in an optically pumped hematite film are dominated by thermal effects beyond 100 ps. The same conclusion was drawn from an oxygen K edge XAS study on CuO,^142^ which found that the time dependence of the thermal response could be well described by thermal diffusion. On femtosecond timescales, the effect of thermal modulation as compared to the electronic response will be less pronounced. However the increase in equilibrium temperature will still be of concern for these measurements, especially for studies employing high repetition rate sources.

A number of experimental techniques can help to reduce pump-induced heating effects. One option is to physically engineer the sample geometry to reduce the temperature rise of the sample due to pumping. Lin *et al.*^143^ employ a gas flow of N2 over the sample to facilitate convective cooling from the sample surface. Application of this technique to PbI_2_ and other thin film has shown significant reduction in the pre-time zero signal due to thermal heating. Another experimental method, which can greatly reduce the amount of sample is rapid raster scanning to effectively increase the area of the pumped volume and reduce the repetition rate of the laser. Jager *et al.* showed that by rotating the sample, the scanning speed was sufficiently high so that each laser shot (repetition rate 100 Hz) exposed a fresh portion of the sample.^144^ This was demonstrated in time-resolved XUV spectroscopy of VO_2_, which undergoes an insulator to metal phase transition at 340 K. In addition to reducing the thermal load on the sample, rapid raster scanning is also advantageous for reducing sample damage. For time-resolved XPS measurements and fluorescence detected XAS, thermal conduction of heat away from the sample can be significantly improved by mounting the thin film on a high thermal conductivity substrate.^138^ This technique is significantly harder to implement for TR-XAS measurements in transmission, using HHG-based sources because any substrate must be thin enough to not significantly attenuate the already limited soft x-ray flux.

**FIG. 5.**
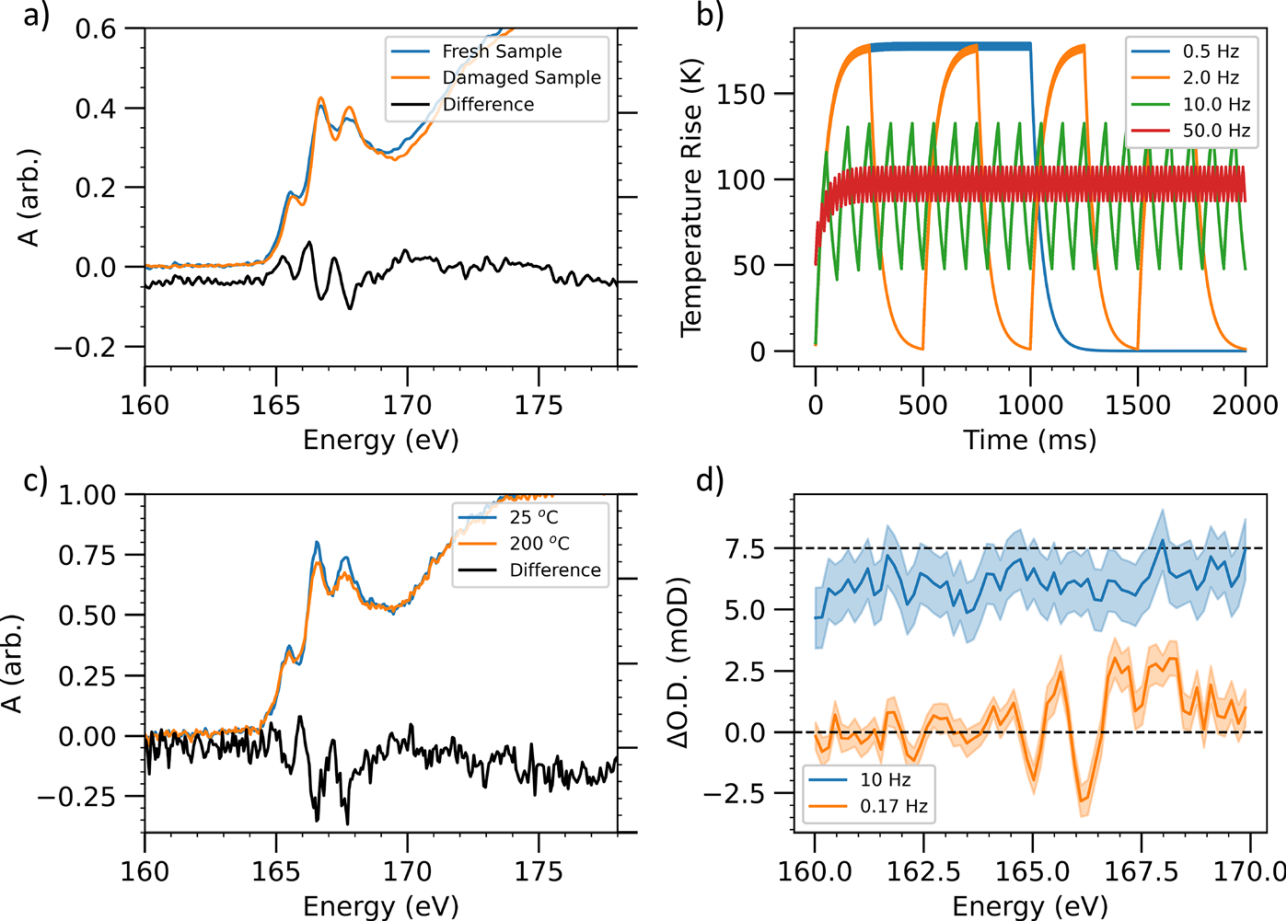
Characterization of the effect of sample damage and heating on P3HT thin films. (a) Sulfur L_2,3_ edge XAS spectra of P3HT films before and after exposure to pump pulses above the damage threshold (fluence 1300 ± 60 *μ*J/cm^2^) for 1 h. The data underlying this figure has been previously published, reproduced with permission from Garratt *et al.*, Nature Commun. **13**, 3414, (2022). Copyright 2022 Authors, licensed under a Creative Commons Attribution (CC BY) license. (b) Sulfur L_2,3_ edge spectrum of P3HT thin films at room temperature and after thermal heating to 200 °C. (c) Simulation of the temperature of a thin-film sample with low thermal conductivity ( γ=0.5) during a pump probe experiment. Sample is exposed to 1 kHz pump pulses, which are switched on and off at four different “acquisition rates” (0.5–50 Hz). (d) Heating-induced pump probe signal at two different data acquisition rates (10 and 0.17 Hz). A 7.5 mOD offset has been applied to the 10 Hz data for clarity. At 0.17 data acquisition rate, a differential signal is observed assigned to thermal heating. Increasing the acquisition rate to 10 Hz reduces the magnitude of the thermal signal below the ∼0.6 mOD noise floor.

While these methods have proven effective at laser repetition rates up to 1 kHz, it is unclear whether they will still be sufficient in future high repetition rate experiments where the thermal load on the sample is increased. In this case, we propose another method to reduce the influence of heating on the transient absorption signal using fast data acquisition, which can be employed in combination with engineering of the sample geometry. During a pump probe experiment, sequential acquisitions with and without the pump beam (termed pumped and unpumped) are recorded sequentially to isolate the change in absorbance due to pumping. For samples with *γ* comparable to $1/f_{\text{rep}}$, the temperature of the sample increases during the pumped acquisition, and the sample cools during the unpumped acquisition. The difference in temperatures therefore manifests as a heating-induced signal, which is typically subtracted. Sufficiently increasing the acquisition rate (defined as $1/(\tau_{p}+\tau_{up})$, where *τ_p_* and *τ_up_* are the pumped and unpumped integration times, respectively) can mean that the sample does not fully reach the equilibrium temperature during the pumped acquisition and does not fully cool during the unpumped acquisition, therefore minimizing the heating-induced signal. This concept is illustrated in Fig. 5 where we consider heating of a low thermal conductivity sample (P3HT) with a 1 kHz pump pulse train at different data acquisition rates. For these calculations we have assumed γ=3.3 s^−1^ and $T_{0}=3.7$ K. This approximates heating of 100 nm thick P3HT films ( $C_{p}=0.7$ Jg^–1^K^–1^) by 550 nm pump pulses at a fluence of 0.8 mJ/cm^2^. At a data acquisition rate of 0.5 Hz, the difference in temperatures between the pumped and unpumped acquisitions is 160° C. Increasing the acquisition rate to 10 or 50 Hz reduces the difference in temperatures to 15 or 2 degrees, respectively, and reduces the average pumped sample temperature from ∼170 to ∼105 °C.

To illustrate the importance of the above-mentioned points, we have characterized the effect of sample heating and damage on thin films of P3HT. As discussed in Ref. 47, the sulfur L_2,3_ edge XAS spectrum of P3HT is not strongly sensitive to photo-excitation of the $\pi \to \pi^{*}$ transition because the three peaks in the spectrum primarily correspond to spin–orbit split S 2p → $\sigma^{*}$(C-S) transitions. However, the S L_2,3_ edge is sensitive to both heating of the sample and optical damage, effects which dominate the pump probe signal at the edge (to within the noise level). Figures 5(a) and 5(b) show the effect of optical damage (a) and thermal heating of the sample (b) on the S L edge spectrum. In both cases, a broadening and reduction of the three pre-edge absorption features is observed. We assign this to a change in film morphology (likely a loss of structural order), with similar microscopic changes in both cases. The heating-induced changes are reversible, while the optical damage is irreversible. Optical pumping of P3HT also produces a broadening of the pre-edge absorption features at the sulfur L_2,3_ edge. This transient absorption signal is delay independent and reversible and is therefore assigned to an increase in equilibrium temperature of the sample by the pump pulse. We have performed a preliminary demonstration of how increasing the rate of data acquisition can reduce the magnitude of this heating-induced signal. Figure 5(d) shows the delay independent signal due to photoexcitation with a fluence of 0.95 mJ/cm^2^ and an average power of 0.75 *μ*W recorded at two data acquisition rates. In the high acquisition rate data, the heating-induced signal is reduced below the ∼1 mOD noise floor of the measurement. This is qualitatively consistent with the significantly reduced average temperature difference (to order 10 °C) at 10 Hz acquisition rate estimated earlier. Further measurements are required to characterize the dependence of the signal on acquisition rate. Modeling of the steady state temperature rise of thin films excited by modulated, pulsed laser sources has been studied in depth due to its importance in time and frequency domain thermoreflectance spectroscopy.^137,145,146^ These more detailed models can be can used to quantitatively predict and minimize the influence of sample temperature modulations on time-resolved x-ray experiments.

## V. CONCLUSION

We have discussed the promising early studies of OPV exciton and charge dynamics using time-resolved soft x-ray probing.^40,47^ These measurements are challenging due to (a) sample heating/damage even at relatively low pumping fluence, (b) propensity for nonlinear effects to manifest even at low pump intensities, (c) the need to carefully tailor the sample thickness to ensure good S/N in optical pump- x-ray probe measurements. We present various methodological strategies to ameliorate these problems, and careful development of these is an important focus of future research. The potential scientific rewards of the measurements, in particular, the prospect to fully track the charge and structural dynamics across the key atomic sites in the materials with few femtosecond resolution, motivate this continued effort. Moreover, new short pulse SXR sources based upon high repetition rate lasers and XFELs will enable far higher sensitivity measurements while permitting the reduction of pumping fluence. HHG-based sources have the advantage of extremely broad bandwidths, which cover the entire water widow, enabling multi-edge x-ray absorption spectroscopy and even time-resolved EXAFS (extended x-ray absorption fine structure) measurements.^147^ The main limitation of these sources is currently the low conversion efficiency and soft x-ray flux, so an increase in the repetition rate of the drive laser will be very beneficial, provided the average SXR flux can also be scaled accordingly. XFEL sources combined with a monochromator upstream of the sample with fluorescence or charged particle detection are better suited to time-resolved XPS measurements and fluorescence detected XAS. These sources can also enable “photon hungry” techniques with increased information content such as time-resolved soft x-ray RIXS on thin-film OPV samples. A major limitation of these sources is the duration of the x-ray pulses (typically 20–50 fs, depending on the spectral resolution of the monochromator) and the challenges associated with co-timing the x-ray pulses with an optical pump with femtosecond accuracy. HHG-based sources provide sub-femtosecond pulses, which are phase-locked to the drive laser field used to generate the pump pulse. Therefore, the timing resolution of TR-XAS measurements with these sources is essentially limited only by the pump pulse duration, and processes on femtosecond to tens of femtosecond timescale can be accessed. Although challenging, the pursuit of measurement of OPV exciton and charge dynamics is motivated by the potential for definitive measurements of critical processes that will aid in future optimization of these materials. The methods developed will also benefit the study of many other functional materials in thin-film formats and even single-layer 2D materials.

## Data Availability

Data sharing is not applicable to this article as no new data were created or analyzed in this study.
